# Genomics of urban adaptation and exaptation in mosquitoes and consequences for vectorial capacity

**DOI:** 10.1016/j.cois.2025.101384

**Published:** 2025-05-08

**Authors:** James E Fifer, Michael Amoa-Bosompem, Dvorah Nelson, Eleanor R Terner, Amel J Clifford, Skylar Tan, Noah H Rose

**Affiliations:** Department of Ecology, Behavior, and Evolution, School of Biology, University of California San Diego, La Jolla, CA 92093, USA

## Abstract

As urbanization accelerates around the world, mosquitoes that are capable of surviving and thriving in urban habitats increasingly spread mosquito-borne diseases. Across the > 3500 known species of mosquitoes, only a few rapidly adapted to the novel (on an evolutionary timescale) urban environments. In this review, we highlight several emerging themes and testable hypotheses from recent literature. First, apparent urban adaptations can be roughly divided into newer adaptations arising in an urban context and exaptations — traits that evolved in a different context, before modern urbanization. Second, variants involved in urban adaptation are often partitioned among species complexes and cryptic lineages, and the history of gene flow–selection balance may be related to the evolution of compact genomic architectures that could facilitate rapid urban adaptation. Third, urban adaptation often has consequences for vectorial capacity — the ability of mosquitoes to serve as effective vectors of a particular pathogen — though the selective drivers and genetic mechanisms underlying these differences are incompletely understood. To fully understand urban adaptation in mosquitoes, we advocate for a coordinated effort to increase linkages between evolutionary ecology, population genomics, and medical entomology research. We discuss the two traits for which all three perspectives are the most developed — host preference and insecticide resistance — before reviewing several other less studied traits.

## Introduction

As urban environments grow around the world, mosquitoes that thrive in these habitats are responsible for an increasing share of mosquito-borne disease transmission. *Aedes aegypti* is a remarkably successful urban invasive, contributing to rapidly expanding burdens of dengue fever [1]. *Culex pipiens var. molestus* thrives in the challenging environment of urban sewers and subways, playing an important role in the urban transmission of the West Nile Virus [2]. Even malaria, historically, a more rural disease, is increasingly spread by urban populations of *Anopheles gambiae* [3] and *Anopheles stephensi* [4]. How are some mosquito populations so successful in urban habitats? As highly diverse species with short generation times, mosquitoes have a tremendous capacity to adapt to novel challenges. However, recent evidence suggests that many urban adaptations in mosquitoes may in fact be exaptations [5]. For example, as we discuss below, while insecticide resistance often evolves in response to contemporary urban pressures, evolution of human host preference seems to predate modern cities. This matters for disease transmission because it suggests that ecological traits relevant to urban adaptation and vectorial capacity may evolve more rapidly than expected from classic theory describing adaptation to truly novel selective pressures. A wide-spread role for exaptation in urban evolution highlights the need to investigate existing phenotypic variation in nonurban populations of potentially dangerous vectors that could facilitate colonization of urban habitats.

## Urban evolution and vectorial capacity

**Figure 1:**
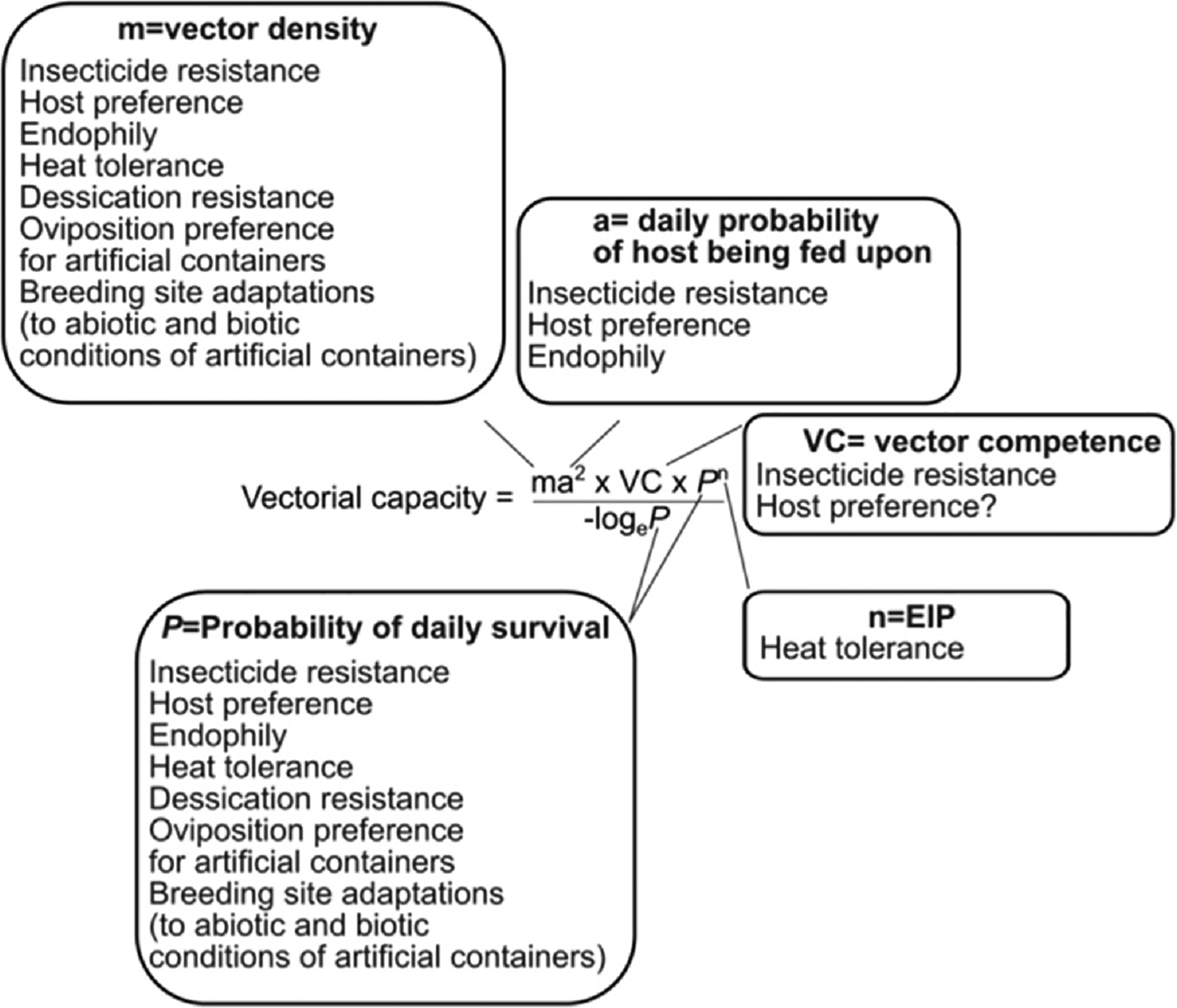
Mosquito urban adaptations (discussed in text) with potential impacts on vectorial capacity.

Vectorial capacity describes the potential of a vector to transmit a pathogen and has been extensively applied to demonstrate the power of mosquitoes as drivers of virus emergence (reviewed in Ref. [6]; Figure 1). Factors that influence vectorial capacity are vector density relative to host (**m**), the daily probability of the host being fed upon (**a**), vector competence (**VC**) — the ability of a vector to transmit a given pathogen given exposure, the probability of daily survival (**P**), and the extrinsic incubation period (**n**) — the time between uptake and ability to transmit the virus. In our overview of mosquito urban adaptations, we use the vectorial capacity formula to frame these adaptations in terms of putative changes in disease transmission.

## Urban evolution of insecticide resistance

Mosquito repellents and insecticides are among the oldest and most effective measures in controlling mosquitoes and the diseases they transmit, but the rapid evolution of insecticide resistance almost invariably follows their heavy use. Although insecticides are widely used in both urban and rural areas, insecticide resistance is particularly prevalent in urban areas, suggesting it plays an important role in urban adaptation [7]. In many cases, urban insecticide resistance appears to be a by-product of resistance to pollution or agricultural runoff, rather than selection from active mosquito control measures (reviewed in Ref. [8]). Insecticide resistance can have strong fitness tradeoffs [9], and resistance alleles are generally quickly lost in the absence of positive selection [10].

The genomic basis of canonical insecticide resistance in mosquitoes appears to be recently evolved, relatively simple, and highly repeatable even across lineages separated for hundreds of millions of years. The biochemical mechanisms by which many mutations in these gene families confer insecticide resistance can be divided into two broad categories, metabolic resistance through detoxification or sequestration of insecticides, and target site insensitivity. There are many examples of *de novo* point mutations or copy number variations affecting the same gene families (e.g. metabolic resistance through cytochrome P450s [11–14] or glutathione transferases [9,13,15–17]) and even the same target gene — most notably, the voltage-gated sodium channel (Vgsc) gene [16,18–20] — across mosquito species. In other cases, introgression (i.e. acquiring genetic variation from another lineage or species) of resistance alleles can explain the repeated evolution of insecticide resistance. For example, while the G119S mutation in the acetylcholinesterase (ace-1) gene evolved independently several times in the *C. pipiens* complex [21], the ace-1 duplication introgressed from *An. gambiae* to *An. coluzzii* [22]. Similarly, the mutant A296S allele, at the resistant to dieldrin (RDL) gene that confers resistance to dieldrin in the *An. gambiae* complex, introgressed from *An. arabiensis* to *An. coluzzii* [22] and the point mutations at Vgsc (F1534C allele) in *Ae. aegypti*’s native range (i.e. Africa) likely introgressed from invasive Asian or American populations [23].

Revisiting this key trait beyond canonical resistance mechanisms yields a more complex picture. First, there are several examples of nonrepeated evolutionary trajectories involving metabolic resistance, since metabolic resistance mutations targeting different genes or gene families can yield similar resistance phenotypes [13]. Second, while insecticide exposure presents a relatively simple selection pressure, exposure itself is diluted or amplified depending on the environmental niche, physiology, and behavior of the organism [24]. *Ae. aegypti* frequently shows stronger target-site resistance to insecticides compared to *Ae. albopictus* from the same region, likely due to increased exposure while seeking hosts indoors [25], raising the question of whether increased exophily could serve as a form of insecticide resistance in endophilic lineages [26]. *An. coluzzii* appears to be more successful than *An. gambiae s.s*. in dense urban areas due to its increased resistance to osmotic stress — a trait that likely arose in the context of ancient niche divergence but contributes as an urban exaptation to other chemical stresses [27]. Intriguingly, *An. stephensi* in Somalia shows resistance to several insecticides in the absence of target-site resistance alleles, potentially reflecting a similar history of niche divergence [28].

**Figure 2:**
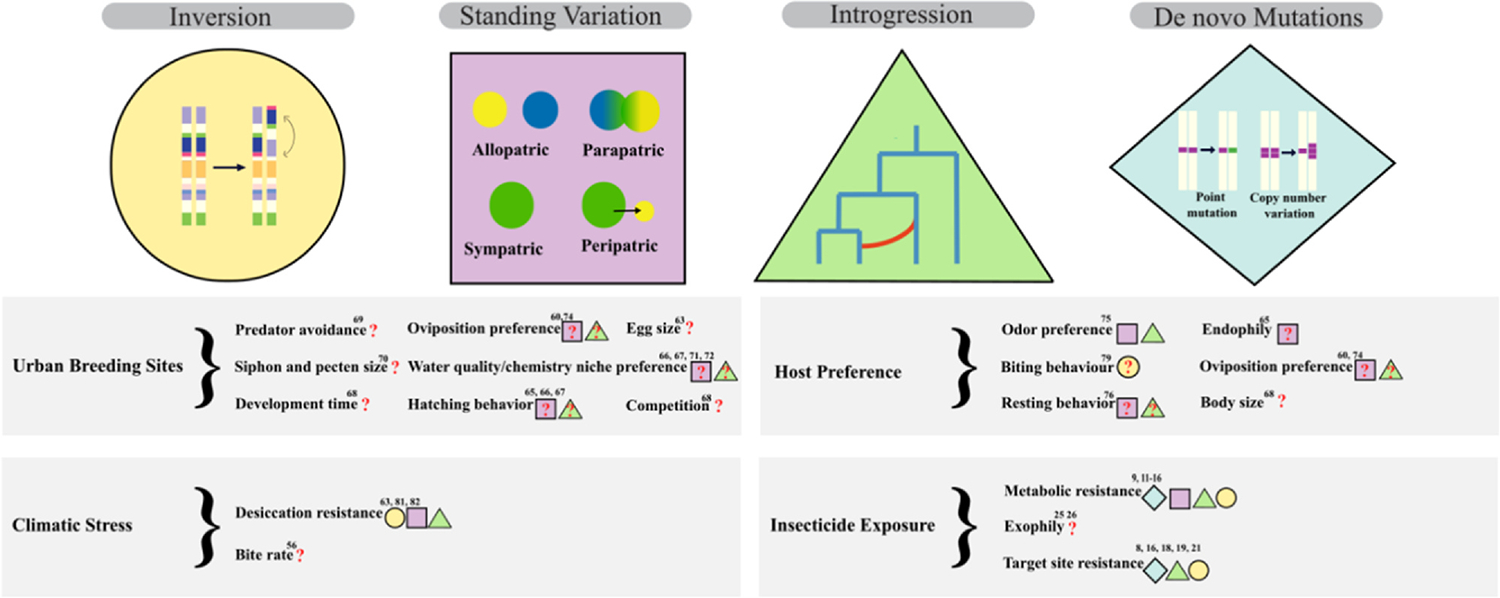
Current knowledge on the types of selective pressures introduced by the urban environment, traits under selection, and underlying genomic architecture. Inversions (yellow circle), standing variation (purple square), introgression (green triangle), and *de novo* mutations (blue diamond), all have evidence for driving urban adaptations. The genomic basis for many traits relevant for urban adaptations has not been examined (these traits are denoted by a red question mark).

The evolution of insecticide resistance increases vectorial capacity by preventing the intended reduction in mosquito densities (**m**) and resulting biting (**a**) (Figure 1). Recent work suggests the evolution of insecticide resistance might also alter *VC* (Figure 1). For example, the lipid transporter Lipophorin (LP) — implicated to at least partially boost plasmodium infection, is upregulated in permethrin-resistant Anopheles females exposed to permethrin as a response to the decrease in fat body abundance [29]. More complex forms of insecticide resistance related to behavioral or ecological divergence could also have important consequences for vectorial capacity. For example, the shift in the period of aggressiveness of some African populations of *Anopheles* from night to evening or early mornings in response to IRS [30] significantly alters their potential as vectors of parasites like *Wuchereria bancrofti* that have peak circulation between 10 pm and 2 am [31]. The cascading impacts of insecticide resistance on other ecologically and vectorially relevant traits as well as the underlying genetic architecture remain an understudied topic and an important avenue for future research (Figure 2).

## Urban evolution of increased preference for humans

Host preference plays an important role in mosquito adaptation to urban environments — mosquito lineages that succeed in urban environments often show a stronger innate preference for biting humans (i.e. anthropophily) compared to nonurban relatives [32,33]. Intuitively, we might expect human host preference to have evolved recently due to low human population densities for much of the mosquito’s evolutionary history, but recent evidence suggests that most human-preferring lineages of mosquitoes are much older than modern cities (Figure 2) [2,34,35]. For example, in the *An. funestus* complex, divergence of the most anthropophilic member *An. funestus s.s*. from the other members of the complex, as well as an introgression event that seems likely to have facilitated its range expansion across Africa, both predate human contact by thousands of years [36]. In other examples, the evolution of anthropophily has required the presence of dense human populations [37], driven by the neolithic transition away from nomadic lifestyles [38] or environmental changes that forced increased contact with humans [34] thousands of years ago. Further divergence within *An. funestus s.s*. is exhibited between indoor and outdoor ecotypes and can be traced to around 1850 years ago, following African rice domestication [35]. Similarly, the loss of tree-hole habitats on the edge of the drying Sahara desert and the emergence of a new ecological niche associated with human water storage coincided with the evolution of the human-specialist form of *Ae. aegypti* [33,34]. This also matches the timing of divergence between forest and urban populations of *An. coluzzii* [39]. The anthropophilic so-called London Underground mosquito, *C. pipiens var. molestus*, appears to also have much older origins, possibly from ancient Egypt [2].

Although it might seem intuitive that anthropophilic lineages would do well in urban habitats, the question remains: why specialize? Nonanthropophilic populations of *Aedes aegypti* are very successful in moderately densely populated towns (< 1000 people per km^2^) across Africa, where they opportunistically feed on humans without becoming specialized on human host odor [33,40]. Similarly, *Ae. albopictus* is an extremely successful urban invasive that regularly bites humans, but host availability seems to be much more important than host preference for determining patterns of host feeding in this species [41]. Despite these observations, the repeated evolution of preference for human hosts across a wide range of mosquito lineages suggests that this trait must be under strong selection in many cases. In particular, the increased preference of *Ae. aegypti* for human hosts in growing African cities, driven by a recent influx of human-specialist ancestry, suggests that the trait is under selection in contemporary urban habitats [33,34]. One possible explanation for the strong selection for specialization could be the effects of sensory tradeoffs involved in identifying human versus nonhuman hosts. Human host odor appears to be an outlier in ‘odor space’, likely due to the relatively unique makeup (e.g. containing squalene and sapienic acid) of our sebum — the protective substance found on human skin and hair [42]. For this reason, the optimal sensory strategy for seeking human hosts may differ substantially from an optimal sensory strategy targeting the shared odor signature of a diverse set of vertebrates. However, further studies are needed to better understand when and why mosquitoes are expected to evolve a strong preference for humans.

How complex is it to evolve a preference for humans, and how repeatable is this evolutionary transition across lineages? Coreceptor mutants for odorant receptor (OR) and ionotropic receptor (IR) gene families in both *Ae. aegypti* and *An. gambiae* suggest that a relatively small number of chemosensory genes play a major role in mosquito host preference (reviewed in Ref. [43]). Likewise, ORs show signatures of selection in anthropophilic lineages of several mosquito families [33,44,45]. These observations might suggest a ceiling on the genetic redundancy for host preference shifts since there are a limited number of genes in the OR and IR gene families. But selection may act on central circuits in addition to peripheral receptors [46], and other sensory inputs beyond odor likely also play an important role [47]. Nevertheless, one of the most exciting prospects for future research in this subfield is the chance to more conclusively determine whether similar changes in peripheral odor sensing are involved in repeated shifts across mosquitoes.

A shift in host preference to humans has serious consequences for disease transmission, not only impacting biting (**a**) but survival (***P***) and density (**m**) (Figure 1) as mosquitoes are able to feed on an abundant host in urban environments. Notably, the geographic distribution of human-specialist *Ae. aegypti* ancestry effectively predicts ZIKV seroprevalence across Africa [48]. Human-specialist ancestry in this species correlates with both an increased probability of biting humans as well as higher mosquito susceptibility to ZIKV [49], suggesting host preference shifts might also impact vectorial capacity through changes in **VC** (Figure 1). However, the mechanisms underlying the latter effect are not known, and analogous effects on dengue virus show complex patterns of strain specificity [50]. While it is not clear if viral adaptation to anthropophilic mosquitoes plays an important role in arboviral emergence [51], mosquitoes shifting to new hosts may exert new selective pressures on the viruses they carry. For example, an increased diversity of WNV in mosquitoes relative to birds may be related to viral lineages from different hosts circulating within mosquito populations, and co-infection leading to the evolution of novel strains [52]. This process of divergent selection introduced by multiple hosts followed by co-infection can lead to novel lineages with outbreak potential, as may have occurred with Oropouche virus [53]. The potential for mosquito evolution to impact viral evolution, and thereby affecting changes in VC, thus represents an exceptionally interesting and important subject for further study.

## Urban adaptations that represent prospects for genomic analysis

**Figure 3:**
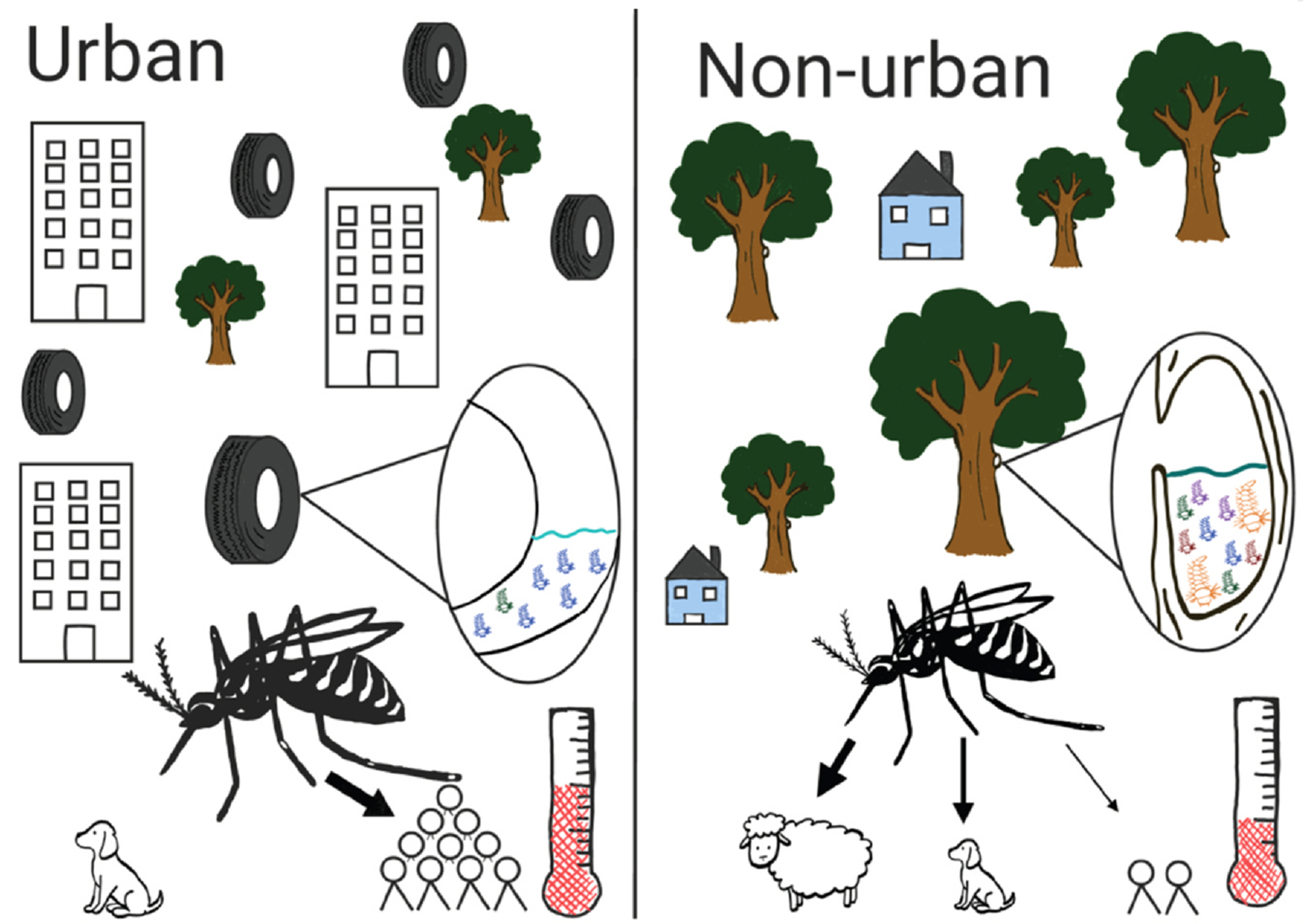
Illustrative potential differences in *Aedes* habitats between urban and nonurban areas, including landscape architecture, the diversity and abundance of available hosts [58], and temperature [59] breeding sites — including water quality [60] competition and predators [61,62].

No two urban environments are identical. Not only do cities vary in population density, infrastructure, climate, and human behavior, but direct control efforts on mosquitoes can impose fluctuating selection pressures (e.g. from the reactive nature of control efforts and funding or policy cycles [54]). Despite the dynamism of urban environments, many selective pressures may be shared across cities (Figure 3). As selection varies across mosquito life stages, it is important to consider adaptations beyond biting adult females to best understand how mosquito populations are evolved to survive in dynamic urban landscapes. There are many different traits that may play a role in urban adaptation, but we have limited information on their genomic architecture and patterns of selection (Figure 2, Table 1).

The various urban adaptations outlined in Table 1 have the potential to modulate the ability of mosquitoes to transmit disease. Under fundamental niche theory, adaptations to an urban environment predict greater population density [55] ***(m)*** in urban areas, a major factor that dictates the vectorial capacity (Figure 1). Additionally, physiological effects due to urban heat islands could impact vectorial capacity by modulating biting patterns ***(b)*** [56] and mosquito–virus interactions ***(n)*** [57] (Figure 1).

## Ancient origins of adaptive variation

Why does it matter that many traits involved in urban adaptation may actually be quite ancient? Over long periods, ecological divergence with gene flow can select for more compact genomic architectures, making it easier for selection to act on linked sets of adaptive variants. Compact genomic architectures hold no discernable competitive advantage when ecological divergence takes place in the presence of geographic barriers. However, in a scenario with high gene flow between populations, a genomic architecture that avoids the separation of locally beneficial alleles will result in individuals with greater local fitness, thereby selecting for genomic islands that are less likely to be broken up by recombination [80]. In both *Ae. aegypti* and *An. gambiae*, this appears to have manifested in the formation of divergent genomic regions that make up relatively small proportions of the genome but have large impacts on the mosquito’s ability to adapt to urban environments and specialize on humans [33,81]. In some cases, chromosomal inversions further enhance this effect, preventing recombination between linked sets of genes involved in adaptation to divergent conditions. In *Anopheles gambiae s.l*., the 2La inversion conveys aridity tolerance, larval thermotolerance, desiccation resistance [82,83], and possibly biting behavior [84] (but see Ref. [85]), suggesting a large role in ecological adaptation. This inversion originated before the split of the *An. gambiae* complex about 2 million years ago [86] and moved between the *An. gambiae* complex and *An. arabiensis* via introgression ~0.5–1 million years ago [86]. There is also emerging evidence for ecologically relevant inversions in *Ae. aegpyti*, which differ in frequency across large geographical regions in Africa [87]. Regardless as to whether the relevant variation is linked through proximity in the genome or by structural variants, the presence of existing older adaptive variation within a species or a species complex that has relevance for modern urban conditions could facilitate rapid evolution of new vector populations to urban environments, as it bypasses the much slower process of waiting for *de novo* mutations to arise [88]. It is perhaps this effect that has led to the abundant examples of exaptations driving success in urban environments across the tree of life [5].

## Figures and Tables

**Table 1:** Traits relevant for urban adaptations and their divergence between species, lineages, and populations. Our criteria for inclusion were any mosquito trait that shows interspecific or intraspecific divergence in relation to urban versus nonurban habitats, or a mosquito trait that, while showing only interspecific divergence (e.g. species/ecotype comparisons: ‘Within genus’ or ‘species A versus species B’) could be relevant in an urban context, as outlined in Figure 3.

Trait	Species/ecotype comparison	Life stage	Authors
Desiccation resistance	*Ae. aegypti* and *Ae. albopictus* versus forest (Ae. *riversi, Ae. galloisi, Ae. flavopietus)* species	Egg	[63]
Larger eggs	*Ae. aegypti* and *Ae. albopictus* versus forest *Aedes* species	Egg	[63]
Erratic hatching after first inundation	*Aedes* versus non-Aedes mosquitoes	Egg	[64]
Higher temperature tolerance	*Ae. aegypti* urban versus forest	Egg	[65]
More hatching in high oxygen water	*Ae. albopictus* urban versus forest	Larva	[66]
More hatching in high oxygen water	*Ae. aegypti* urban versus forest	Larva	[67]
Competitive advantage with scarce food pulses	*Ae. aegypti* domestic versus forest	Larva	[68]
Competitive advantage abundant food pulses	*Ae. aegypti* forest versus domestic	Larva	[68]
Predator avoidance	*C. pipiens* versus *Ae. aegypti*	Larva	[69]
Slower development	*Ae. aegypti* domestic versus forest	Larva	[68]
Smaller siphones and larger pecten spines	*C. molestus* versus *C. pipiens*	Larva	[70]
Pollution tolerance	*C. molestus* versus *C. pipiens*, within *Anopheles*	Larva	[71,72]
Flushing avoidance	*Ae. aegypti* versus *C. pipiens*	Pupa	[73]
Oviposition preference for artificial containers	*Ae. aegypti* urban versus forest	Adult	[60,74]
Endophily (house entering)	*An. gambiae, An. arabiensis*, and *A. quadrimaculatus* versus other *Anopheles*	Adult	[75]
Resting behavior variation	Within *Anopheles*	Adult	[76]
Higher activity during the day	*Ae. aegypti* urban versus forest	Adult	[77]
Sleep and activity patterns	*Ae. albopictus* populations from greater human density urban/suburban areas	Adult	[78]
Larger body size	*Ae. aegypti* domestic versus forest	Adult	[68]
Higher feeding aggression	*Ae. triseriatus* urban versus forest	Adult	[79]

## Data Availability

No data were used for the research described in the article.
